# Pregnant mice lacking indoleamine 2,3‐dioxygenase exhibit preeclampsia phenotypes

**DOI:** 10.14814/phy2.12257

**Published:** 2015-01-19

**Authors:** Mark K. Santillan, Christopher J. Pelham, Pimonrat Ketsawatsomkron, Donna A. Santillan, Deborah R. Davis, Eric J. Devor, Katherine N. Gibson‐Corley, Sabrina M. Scroggins, Justin L. Grobe, Baoli Yang, Steven K. Hunter, Curt D. Sigmund

**Affiliations:** Department of Obstetrics & Gynecology, University of Iowa, Iowa City, Iowa; The Center for Hypertension Research, University of Iowa, Iowa City, Iowa; Department of Pharmacology, University of Iowa, Iowa City, Iowa; Department of Pathology, University of Iowa, Iowa City, Iowa

**Keywords:** Animal model, indoleamine 2,3‐dioxygenase, preeclampsia, T cell

## Abstract

Preeclampsia is a cardiovascular disorder of late pregnancy that is, commonly characterized by hypertension, renal structural damage and dysfunction, and fetal growth restriction. Prevailing etiologic models of this disorder include T‐cell dysfunction as an initiating cause of preeclampsia. Indoleamine 2,3‐dioxygenase (IDO), an enzyme that mediates the conversion of tryptophan to kynurenine, has been linked to preeclampsia in humans, and is known to regulate T‐cell activity and an endothelial‐derived relaxing factor. To test the hypothesis that IDO is causally involved in the pathogenesis of preeclampsia, mice deficient for IDO (IDO‐KO) were generated on a C57BL/6 background. IDO‐KO and wild‐type C57BL/6 mice were bred, and preeclampsia phenotypes were evaluated during pregnancy. Pregnant IDO‐KO mice exhibited pathognomonic renal glomerular endotheliosis, proteinuria, pregnancy‐specific endothelial dysfunction, intrauterine growth restriction, and mildly elevated blood pressure compared to wild‐type mice. Together these findings highlight an important role for IDO in the generation of phenotypes typical of preeclampsia. Loss of IDO function may represent a risk factor for the development of preeclampsia. By extension, increased IDO activity, reductions in IDO reactants, or increases in IDO products may represent novel therapeutic approaches for this disorder.

## Abstract (precis)

Indoleamine 2,3 Dioxygenase (IDO) has been implicated in the T‐cell dysregulation and early pathogenesis of the devastating pregnancy‐related hypertensive disease, preeclampsia. We demonstrate that chronic deficiency in IDO in a IDO‐KO mouse model recapitulates many of the essential phenotypes of human preeclampsia including renal damage. This novel model will help dissect the role of IDO and T cells in the early pathogenesis of preeclampsia.

## Introduction

Preeclampsia is a devastating cardiovascular disorder of late pregnancy that affects roughly 5% of all pregnancies and results in the death of 76,000 mothers and 500,000 babies each year (Kuklina et al. 2009). Even if they survive the preeclamptic pregnancy, both mothers and children are still predisposed to cardiovascular and metabolic disease for the remainder of their lives (Godfrey 2002; Swamy et al. 2008).

Many studies support the concept that an immune system response to the pregnancy triggers the cascade of physiologic effects that ultimately result in preeclampsia. Multiparous women who conceive by the same partner are at a lower risk for developing preeclampsia, suggesting that a tolerance to a paternal antigen develops as a result of a previous pregnancy (Need 1975; Saftlas et al. 2003). In preeclamptic women, there is also an increased expression of inflammatory cytokines (Chen et al. 1996). Animal studies similarly support an immune role in preeclampsia, as transfer of activated Th1‐like cells can lead to the development preeclamptic phenotypes in pregnancy in mice (Zenclussen et al. 2004). One theory holds that the immune response to the fetus results in damage to the vascular endothelium, resulting in preeclampsia (Redman et al. 1999). One candidate immunogenic protein that may mediate such a mechanism is indoleamine 2,3‐dioxygenase (IDO).

Indoleamine 2,3‐dioxygenase is a cytosolic hemoprotein widely distributed in mammalian tissues. It is a critical enzyme in T‐cell‐mediated immune responses, as it catalyzes the rate‐limiting step of tryptophan (Trp) catabolism that is, necessary for T‐cell function. T‐cell proliferation is inhibited in vitro by rapid Trp depletion, with arrest at the mid‐G1 point in the cell cycle (Munn et al. 1996, 1999). Santoso et al. (2002) demonstrated that significantly less IDO was expressed in the endothelium of placentae of term preeclamptic women. Munn et al. (1998) found that exposure to 1‐methyl‐tryptophan (1‐MT), an IDO inhibitor, resulted in hemorrhage, inflammation, degeneration, and death of concepti from allogeneic crosses, whereas syngeneic embryos were unaffected. Other studies have shown that IDO+ cells are present at the maternal–fetal interface soon after implantation (Kamimura et al. 1991; Tatsumi et al. 2000; Mellor and Munn 2001; Norwitz et al. 2001; Suzuki et al. 2001), and decreased IDO activity and mRNA have been detected in preeclamptic placentae (Kudo et al. 2000, 2003). Coupled with an elevated maternal plasma Trp, there is decreased catabolism of Trp due to decreased IDO activity in preeclamptic placentas (Kudo et al. 2003). Reduced IDO activity is associated with increased T‐cell infiltration and increasing severity of preeclampsia (Nishizawa et al. 2007). Collectively these findings support the hypothesis that IDO tonically inhibits an immunologic reaction against the feto‐placental unit and thereby prevents preeclampsia (Hunter et al. 2000).

Indoleamine 2,3‐dioxygenase may also play a role in the oxidative damage to the endothelium typical of preeclampsia (Hubel 1999; Roberts and Hubel 1999; Myatt et al. 2000). IDO utilizes 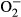 radicals to catabolize L‐tryptophan (Thomas and Stocker 1999). Reduced activity of IDO in preeclampsia may increase bioavailable 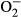 thus contributing to the oxidative damage in preeclampsia. We therefore hypothesize that the reduced levels of IDO in an IDO knockout mouse model recapitulates essential phenotypes of human preeclampsia.

## Materials and Methods

### Generation of the IDO‐KO mouse model

**Figure 1.**
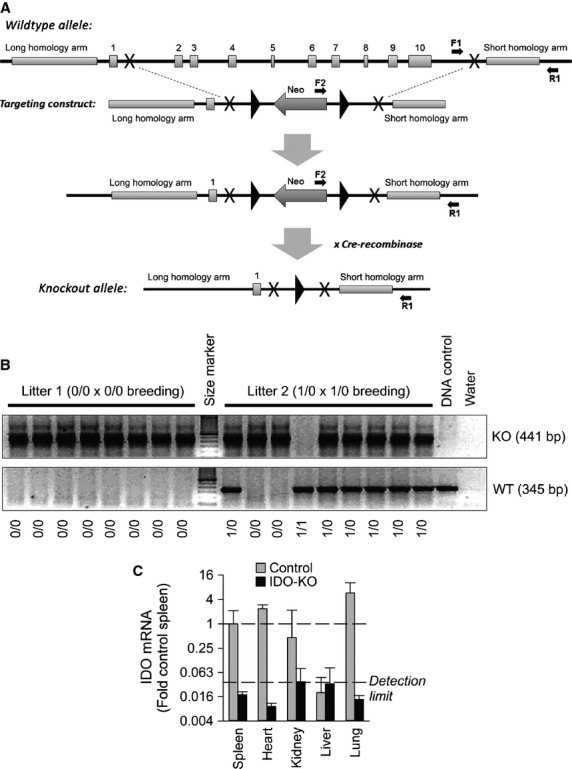
Generation and validation of the IDO‐KO mouse model. (A) Schematic illustrating the IDO gene targeting strategy. (B) PCR amplification of DNA samples from mice of various genotypes. (C) RT‐PCR amplification of IDO mRNA in selected tissues from control and IDO‐KO mice. Control *n* = 3, IDO‐KO *n* = 3.

IDO‐KO mice were generated utilizing Cre‐Lox recombination. The endogenous mouse IDO gene has 10 exons, spanning 15 Kb. The translation start code is located in exon 1 and the stop codon is located in exon 10. To generate the KO mice, a 5 Kb fragment was amplified, containing exon 1, as the long arm (LA) or the upstream homology, and a 1 Kb fragment, downstream from exon 10 as the short arm (SA) or the downstream homology. The total homologies were 6 Kb and the deleted region after homologous recombination is 11.7 Kb. As shown in Figure 1A, the majority of the coding region was deleted in the KO allele with 375 of 408 amino acids being deleted from the protein. Exon 1 was the only intact exon, and it codes for 33 amino acids. Both fragments were subcloned into pOSdupdel, with a Neo gene in the opposite orientation. Targeted embryonic stem (ES) cells were screened with PCR using the following three primers: F1: AAATGAGTTGTGAAACCAATCATGG, R1: AAAGAGGAGCTGGAGGACT AATGG, and F2: TGTCTGTTGTGCCCAGTCATAGC. F1‐R1 amplifies a band of 1401 bp from the wild‐type allele, whereas F2‐R1 amplifies a band of 1807 bp from the KO allele. Positive ES clones were obtained and one was used to generate chimeras and subsequently allow germline transmission. Mice are genotyped using the following three primers: CL108F1, 5′ ATTGTTTGCCAGCCTTAGCA 3′, CL108F2: 5′ GGAGAGGCTTTTTGCTTCCT 3′, and CL108R: 5′ CCCCAGAGAGGGTGTCTGTA 3′. These primers yield the WT allele of 345 bp and the KO allele of 441 bp. Mice were backcrossed 12 generations onto the C57BL/6 background.

### Timed breeding

Female mice from 3 to 6 months of age were mated with corresponding, age‐matched syngeneic males (“control” pregnancies = C57BL/6 male × C57BL/6 female, whereas “IDO‐KO” pregnancies represent IDO‐KO male × IDO‐KO female). Gestational day (GD) 0.5 was defined as the day a vaginal plug was detected.

### Blood pressure telemetry

Telemeters (Data Science International, St. Paul, MN) were implanted in a subset of female mice using our previously published methodology (Lavoie et al. 2006). In pregnant mice, telemeters were implanted at GD7 in C57BL/6 and IDO‐KO dams. Data from the telemeters were recorded for 30 sec every 5 min from GD8 through GD17. A cohort of nonpregnant C57BL/6 females was also implanted with telemeters and pressures were recorded after implantation and recovery, during the same time period as the pregnant cohort. For implantation, mice were anesthetized with ketamine/xylazine (87.5 mg and 12.5 mg/kg); the catheter was placed into the left common carotid artery, and the transmitter was placed subcutaneously along the left flank. Data were collected and stored using Dataquest A.R.T. (Data Science International, St. Paul, MN).

### 24‐h urine protein concentration measurement

On GD17, all pregnant dams were placed into single‐mouse‐sized metabolic chambers (Nalgene) to collect urine for 24 h. The urine collection cup was suspended in ice throughout the time of urine collection to retard protein degradation. At 24 h, the urine was collected, the volume was measured, and then it was flash frozen in liquid nitrogen for future total protein concentration measurement. Total protein measurement was performed according to the manufacturer's protocol utilizing a commercially available bicinchoninic acid (BCA) colorimetric total protein assay kit (Thermo Scientific, Rockford, IL).

### Necropsy and kidney histology

All dams were killed using a lethal dose of intraperitoneal phenobarbital (50 mg/mouse) at GD18. The total number and masses of individual fetuses were recorded. Maternal kidneys were collected for histology. A subset of kidneys were immersion fixed in 10% neutral‐buffered formalin for light microscopy, whereas others were sectioned fresh into 1‐mm slices and immersed in 2.5% EM grade glutaraldehyde in 0.1 mol/L cacodylate buffer for approximately 48–72 h. Formalin‐fixed kidney samples were routinely processed, embedded in paraffin, and sectioned at 4 *μ*m thickness. Routine hematoxylin and eosin stains were performed as well as methenamine Periodic acid‐Schiff (PAS) to assess glomerular basement membrane thickness. Gluteraldehyde‐fixed kidney samples were processed using a Leica EM AMW autoprocessor and embedding in epoxy resin. 1 *μ*m thick sections were cut with a Leica EM UC6 ultramicrotome, stained with toluidine blue and examined under a light microscope. Thin sections were cut from blocks and placed on nickel grids. These were then stained with 5% uranyl acetate and lead citrate for contrasting and viewed using a JEOL JEM‐1011 transmission electron microscope. All kidney processing and histology was performed by Comparative Pathology staff as previously (Santillan et al. 2014).

### Vascular function assessment

In a separate cohort of 3–6‐month‐old syngeneically mated C57BL/6 control and IDO‐KO dams and a cohort of nonpregnant C57BL/6 and IDO‐KO females were utilized for vascular reactivity experiments in aortas and mesenteric arteries according to our previously published methods (Halabi et al. 2008; Ketsawatsomkron et al. 2012). Briefly, pregnant mice at GD18 and nonpregnant mice were killed via phenobarbital overdose. The entire aorta was dissected to the level of the iliac arteries and divided into four equal segments of 4–5 mm in length. The aortic segments were placed in Krebs buffer (NaCl 118.3, KCl 4.7, CaCl_2_ 2.5, MgSO_4_ 1.2, KH_2_PO_4_ 1.2, NaHCO_3_ 25, glucose 11 mmol/L). Connective tissue and adventitial fat were removed. Each aortic ring segment was suspended in an organ bath containing 20 mL Krebs solution maintained at 37°C and were connected to a force transducer via steel hooks to measure isometric tension. Resting tension was increased stepwise to reach 0.5 g, and the rings were allowed to equilibrate for 45 min. An initial 100 mmol/L maximal potassium chloride (KCl) contraction curve was performed and relaxed with sodium nitroprusside (SNP). After equilibration, precontraction (40–50% of max) with PGF2*α* (5–10 mmol/L) was performed. After a stable contraction plateau was reached, dose–‐response curves were obtained for acetylcholine (ACH; 0.01–30 mmol/L) and SNP (0.01–30 mmol/L). Data were collected with a PowerLab/8SP and analyzed with associated Chart 5 software (AD Instruments, Colorado Springs, CO).

Concurrently, second‐order mesenteric arteries were dissected and placed in Krebs buffer. Arteries were transferred to a pressurized myograph system (Danish Myo Technology, Ann Arbor, MI) and equilibrated for 30 min at 75 mmHg under no‐flow conditions. Lumen and external diameter under passive conditions at a pressure of 75 mmHg were utilized for structural analyses (wall thickness, % media/lumen ratio and cross‐sectional area; CSA). Dose–response curves were performed using ACH and SNP as previously (Ketsawatsomkron et al. 2012).

### Statistical analysis

All data are expressed as mean ± SEM. Data were excluded only when values were outside the mean ± 2 SD range. Multiple comparisons were made by two‐way repeated‐measures ANOVA, and post hoc comparisons were performed via Tukey multiple comparisons procedures, using SigmaStat (Systat Software, Inc. San Jose, CA). Two‐sided Student's *t*‐test was used where appropriate. A *P*‐value < 0.05 was considered significant.

## Results

Successful generation of IDO‐KO mice was confirmed by multiple methods. First, PCR‐based genotyping of mice highlights expected, atypical PCR products in mice with gene disruption (Fig. 1B). Gene disruption in IDO‐KO mice and normal tissue‐based expression patterns in control mice were then confirmed at the mRNA level by quantitative RT‐PCR in all maternal tissues and the placentae (Fig. 1C).

**Figure 2.**
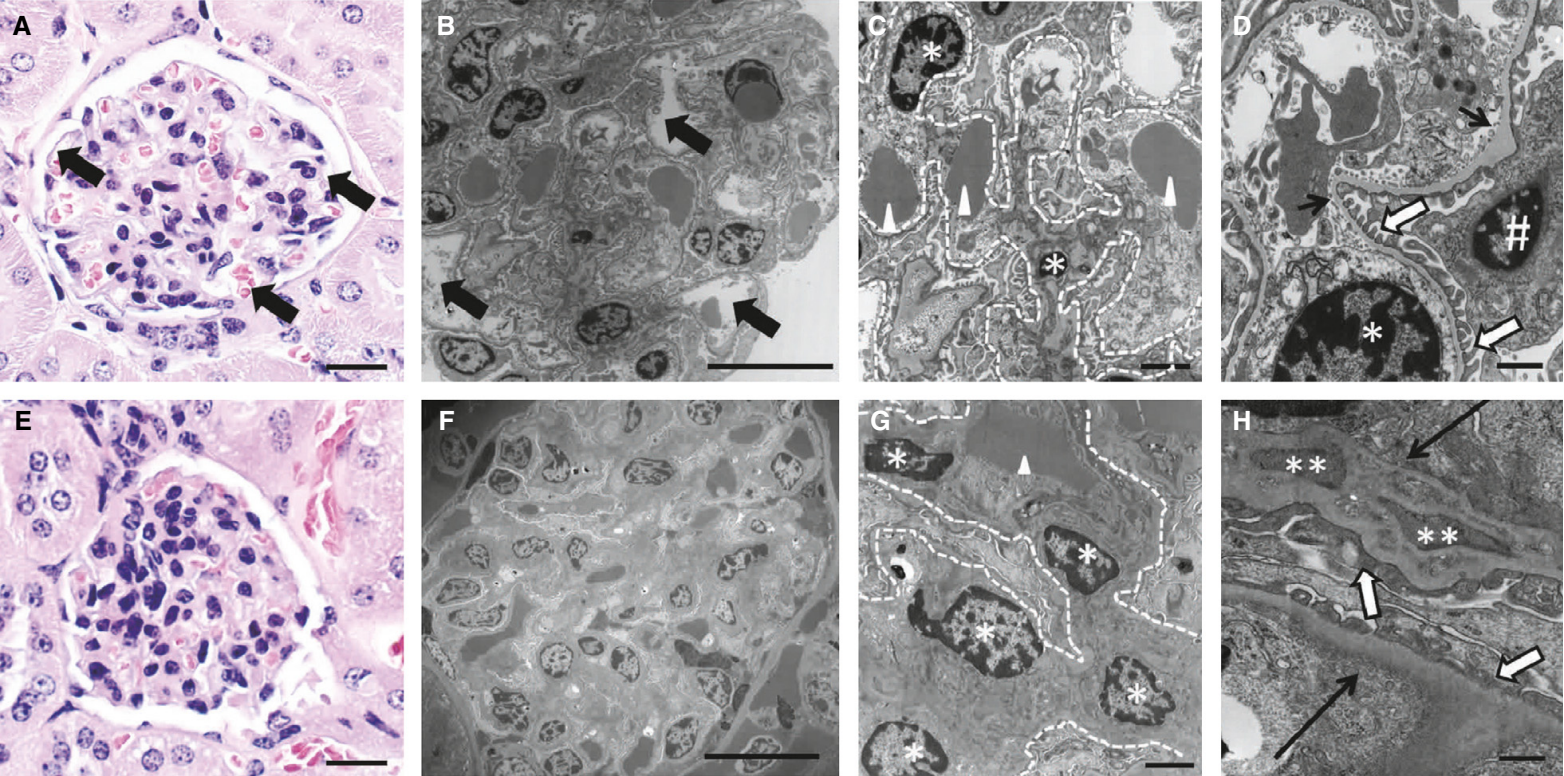
Disruption of IDO alters maternal kidney structure. (A, B) Glomeruli from wild‐type mice. Arrows point to open capillary loops in the glomerular tufts. H&E stain, bar = 20 *μ*m; Transmission electron microscopy (TEM), bar = 10 *μ*m. (C) Higher magnification of wild‐type glomerulus. Arrowheads point out single red blood cells within glomerular capillaries. Stars highlight the nuclei of endothelial cells and the dotted lines outline the glomerular capillary loops. TEM, bar = 2 *μ*m. (D) Highest magnification of wild‐type glomerulus. Thin black arrows point out the glomerular basement membrane, whereas white arrows highlight podocyte foot processes. Stars again indicate endothelial cell nuclei while # demarks the podocyte nucleus. TEM, bar = 500 nm. (E, F) Glomeruli from IDO‐KO mice. Note the lack of open capillary loops in the glomerular tufts. H&E stain, bar = 20 *μ*m; TEM, bar = 10 *μ*m. (G) Higher magnification of the IDO‐KO glomerulus. A single red blood cell is present (arrowhead) and the capillary space, as indicated by the white dotted lines, is expanded and filled with swollen endothelial cells (stars). TEM, bar = 2 *μ*m. (H) Highest magnification of IDO‐KO glomerulus. The basement membrane of this capillary (thin black arrow) is thickened by electron‐dense deposits (double stars). Podocyte foot processes also exhibit mild foot process fusion in some animals. TEM, bar = 2 *μ*m.

**Figure 3.**
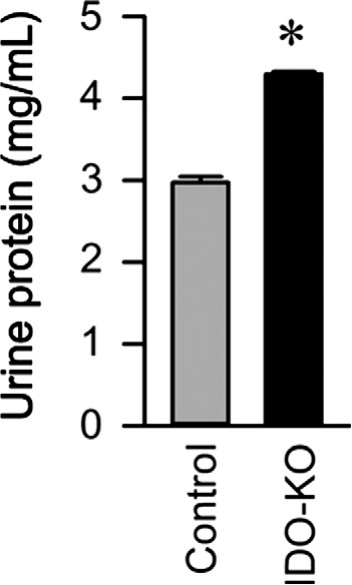
Disruption of IDO causes proteinuria. 24‐h urine protein detected by BCA assay. Control *n* = 4, IDO‐KO *n* = 4. Data presented as mean ± SEM. *P < 0.05.

IDO‐KO dams exhibited significant renal pathology and dysfunction at the end of gestation. Both H&E staining and electron microscopic examination of the kidney revealed pathognomonic glomerular endotheliosis in IDO‐KO mice (Fig. 2). In addition, analysis of urine collected on GD17 uncovered a robust (~50%) increase in urine protein (Fig. 3). Together these data highlight the generation of preeclampsia renal phenotypes with the loss of IDO in mice.

**Figure 4.**
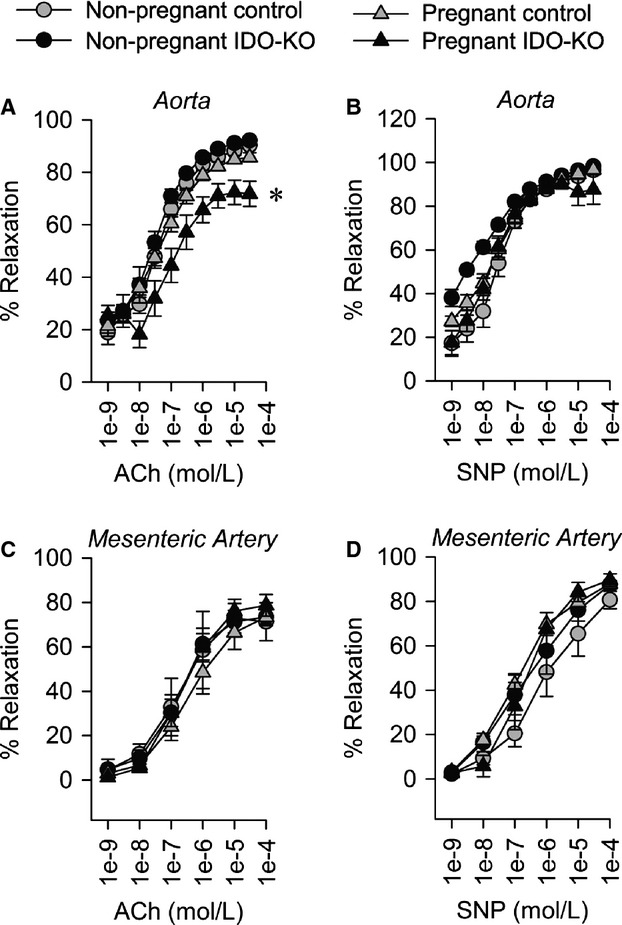
Disruption of IDO causes maternal vascular dysfunction. (A) Aortic relaxation to acetylcholine. (B) Aortic relaxation to sodium nitroprusside. (C) Relaxation of second‐order branch mesenteric artery to acetylcholine. (D) Relaxation of second‐order branch mesenteric artery to sodium nitroprusside. Pregnant Control *n* = 10, Pregnant IDO‐KO *n* = 7, Nonpregnant Control *n* = 5, Nonpregnant IDO‐KO *n* = 4 Data presented as mean ± SEM. *P < 0.05.

Pregnancy‐specific endothelial dysfunction was specifically observed in conduit arteries (aorta) of IDO‐KO mice. Aortae from pregnant and nonpregnant control and nonpregnant IDO‐KO mice exhibited normal relaxation responses to both ACH and SNP. In contrast, aortae from pregnant IDO‐KO mice exhibited significantly impaired relaxation to ACH but normal relaxation response to SNP, highlighting both a pregnancy‐ and endothelial‐specific change in aortic function (Fig. 4A and B). In contrast to aortic function, second‐order branches of mesenteric arteries from all treatment groups exhibited normal relaxation responses to both ACH and SNP (Fig. 4C and D). Together these data highlight a role for IDO in the control of endothelial function that is, specific to pregnancy and vessel type.

**Figure 5.**
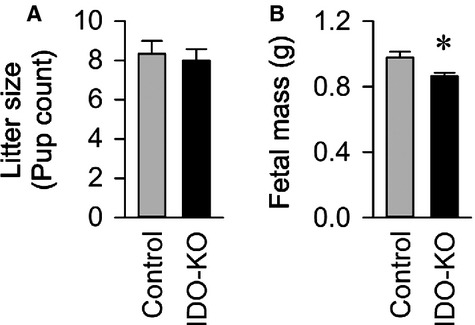
Disruption of IDO causes decreases fetal mass. (A) Average litter size per mother. (B) Average mass of individual fetuses. Control *n* = 50 fetuses from *n* = 6 dams, IDO‐KO *n* = 48 fetuses from *n* = 6 dams. Data presented as mean ± SEM. *P < 0.05.

Disruption of IDO suppressed intrauterine growth, but not fecundity. The number of fetal‐placenatal units per pregnancy (Fig. 5A) and placental weights were comparable between IDO‐KO and control dams (data not shown), but individual fetuses from IDO‐KO dams were significantly smaller than fetuses from control pregnancies (Fig. 5B). On histological examination, IDO deficiency revealed no significant differences in the thicknesses of the labyrinth and spongiotrophoblast layers compared to control placentae based on H&E staining (data not shown). These data are again consistent with a role for IDO disruption in the generation of preeclampsia phenotypes, including intrauterine growth restriction.

**Figure 6.**
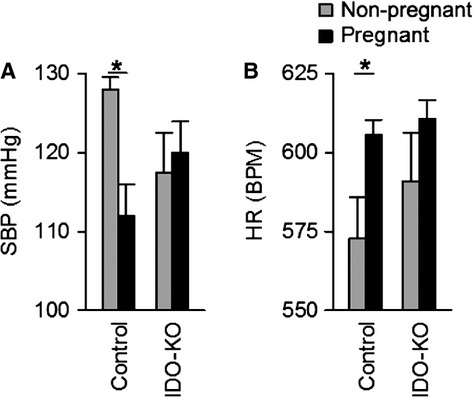
Disruption of IDO has minimal effects on maternal blood pressure during pregnancy. (A) 24‐h average systolic blood pressure and 24‐h average heart rate (B) in IDO‐KO and control dams were averaged across GD9 through GD12. IDO KO mice do fail to exhibit a pregnancy‐induced decrease in blood pressure. Nonpregnant 24‐h average systolic blood pressure and 24‐h heart rate were determined on day 8 post implantion of the telemeter. Control nonpregnant *n* = 4, Control pregnant *n* = 5, IDO‐KO nonpregnant *n* = 3, IDO‐KO pregnant *n* = 6. Data presented as mean ± SEM. *P < 0.05.

Blood pressure is typically reduced during pregnancy in wild‐type animals, and this stereotyped reduction was observed in control mice (Fig. 6A). This normal pregnancy‐induced reduction in blood pressure was not observed in the IDO‐KO mice. Blood pressures of pregnant IDO‐KO mice were intermediate between nonpregnant and pregnant control mice and was not significantly different from either group. Clearly, overt hypertension during pregnancy was not noted in IDO‐KO mice. Heart rate, which is typically increased during pregnancy in response to reduced blood pressure, was similarly elevated during pregnancy more in control than IDO‐KO animals (Fig. 6B). Together these data illustrate that despite evidence of other phenotypes consistent with preeclampsia, disrupting IDO had a minimal effect upon blood pressure during pregnancy.

## Discussion

This study illustrates four key findings. First, genetic disruption of the IDO gene is sufficient to precipitate multiple phenotypes of preeclampsia in pregnant mice, including renal histological pathology and dysfunction, aortic endothelial dysfunction, and intrauterine growth restriction. Second, genetic disruption of the IDO gene is not sufficient in isolation to cause robust increases in late‐pregnancy blood pressure, as are observed in pregnant women and other animal models of preeclampsia. Third, disruption of the IDO gene causes intrauterine growth restriction, though this study design precludes distinguishing the effects of loss of IDO in the dam versus the fetus as all KO matings were syngeneic (KO × KO). Fourth, disruption of the IDO gene has no obvious effect upon maternal fecundity or fetus survival. In aggregate, these findings demonstrate that genetic disruption of the IDO gene is sufficient to partially phenocopy preeclampsia in mice, and therefore these findings are supportive of a role for IDO in the pathogenesis of preeclampsia.

Importantly, our data differs from other previously published studies regarding the consequence of suppressed IDO activity during pregnancy. Both Munn et al. and Nishizawa et al., utilized a pharmacologic inhibition of IDO by acute administration of 1‐methyl‐tryptophan (1‐MT), an IDO inhibitor. In contrast to our data, Munn demonstrated a significant difference in fetus size *and* litter size. These differences may be attributable to the manner in which IDO deficiency is induced. An acute administration of 1‐MT may cause a significant change in embryo implantation, for example. In contrast, with chronic IDO deficiency due to genetic disruption, there may be other chronic compensatory mechanisms that could protect embryo during implantation. Although Munn did not investigate blood pressures, Nishizawa et al. utilized a similar pharmacologically inhibited IDO model and demonstrated a modest increase in blood pressure with IDO inhibition. Again, our data are partially supportive of this effect but compensatory changes occurring due to chronic loss of IDO may contribute to a blunted phenotype in our study. Despite the possibility of compensatory changes with genetic IDO disruption, one strength of our study is that we were able to demonstrate global IDO deficiency in our model. In the pharmacologic model, IDO functional activity was not assayed in multiple tissue types and it is unclear whether tissue‐specific access of the inhibitor or the effects of the inhibitor may contribute to differences in results among our studies. We contend that a genetic model of preeclampsia may prove to be more physiologically relevant, as functional differences in IDO in humans are likely to be chronic and genetically based as opposed to acute and environmentally based. Regardless, our data and those of Munn and Nishizawa agree that IDO appears to be mechanistically linked to preeclampsia.

Our data add to the growing collection of observations that support an immune, rejection‐like response to the feto‐placental unit as the root cause of preeclampsia. Among these observations is the finding that multiparous women who conceive by the same partner are at lower risk for developing preeclampsia (Need 1975; Dekker and Robillard 2003; Saftlas et al. 2003). Because different paternity is associated with an increased risk to that of repeated paternity, this finding suggests that a tolerance to the paternal antigen develops as a consequence of a prior pregnancy (Robillard et al. 1999). T‐cell responses to such are believed to be an important etiology of preeclampsia. Zenclussen and colleagues transferred activated Th1‐like cells into pregnant mice causing the development of preeclamptic symptoms such as elevated blood pressure and proteinuria (Zenclussen et al. 2004). Notably, these findings only developed when cells were transferred into pregnant, but not nonpregnant, mice. A maternal T‐cell reaction against paternal antigens can be also be induced in mice when IDO is pharmacologically inhibited (Munn et al. 1998), resulting in fetal rejection (Mellor et al. 2001).

Consistent with the concept that reduced IDO is detrimental, Santoso et al. (2002) demonstrated that significantly less IDO was expressed in placentae of term preeclamptic women than in control placentae. Liu et al. correlated reduced IDO levels to reduced Foxp3 expression in placentae from preeclamptic women and suggested that loss of fetal tolerance in preeclampsia is due to reduced T regulatory cells, however, upon preliminary analysis we observed no significant difference in Foxp3 expression via qPCR in IDO‐KO versus control placentae (IDO‐KO *n* = 5: 22.5 ± 0.39, vs. control *n* = 6: 24.3 ± 1.2, *P* = 0.2201; Liu et al. 2011). These data suggest that the Treg compartment is likely to be intact, however, future studies will examine the possibility of decreased Treg function (suppressive activity) versus reduced Treg numbers in IDO‐KO mice (Sharma et al. 2007; Baban et al. 2009). Finally, depleting T cells of tryptophan inhibits their expansion due to an arrest in the cell cycle at the G1 phase, providing some hints at the molecular mechanisms through which IDO could contribute to T‐cell function (Taylor et al. 1996; Munn et al. 1999; Kudo et al. 2003). Thus, IDO appears to be important for normal T‐cell function, and compromised T‐cell function (possibly through disruption of IDO) may represent a major contributor to the initiation of preeclampsia. Future studies with this model will include allogeneic crosses and other inducers of immunologic function.

In summary, our data demonstrate that genetic disruption of IDO is sufficient to induce selected phenotypes of preeclampsia in mice. These data complement previous studies using pharmacological inhibitors of IDO, and support a role for the immune system, possibly involving T cells, in the pathogenesis of preeclampsia. Future studies investigating the effects of stimulating IDO, reducing its reactants or increasing its products, are required to determine whether such manipulations would be beneficial in the prevention or treatment of preeclampsia.

## Acknowledgments

The authors thank the University Of Iowa Office Of Animal Resources and the staff of the University of Iowa Transgenic and Genome Manipulation Facility for their assistance.

## Conflicts of Interest

None declared.
